# Addressing non-communicable diseases risk factors through innovative and context-sensitive approaches in the eastern Mediterranean region: a systematic review

**DOI:** 10.3389/fpubh.2026.1833047

**Published:** 2026-05-28

**Authors:** Mohammed Elmadani, Godfrey Mbaabu Limungi, Mohammed Mustafa, Dahabo Galgalo, Maha Besbes, Evans Kasmai Kiptulon, Peter Onchuru Mokaya, Melese Dereje Mesfi, Eltagi Elsadig, Simon Klara, Hussain Alizadeh, Adrienn Ujváriné Siket, Gabriella Hideg-Fehér, Viktória Prémusz, Orsolya Máté

**Affiliations:** 1 Doctoral School of Health Sciences, Faculty of Health Sciences, University of Pécs, Pécs, Hungary; 2 Department of Epidemiology, Faculty of Public Health, University of El Imam El Mahdi, Kosti, Sudan; 3 Jamhuriya Research Centre, Jamhuriya University of Science and Technology (JUST), Mogadishu, Somalia; 4 Faculty of Nursing Sciences, University of El Imam El Mahdi, Kosti, Sudan; 5 Division of Hematology, First Department of Internal Medicine, Medical School, University of Pécs, Pécs, Hungary; 6 Doctoral School of Health Sciences, Faculty of Health Sciences, University of Debrecen, Debrecen, Hungary; 7 Institute of Emergency Care, Pedagogy of Health and Nursing Sciences, Faculty of Health Sciences, University of Pécs, Pécs, Hungary

**Keywords:** community-based interventions, digital health, eastern Mediterranean region, NCD risk factors, non-communicable diseases (NCDs)

## Abstract

**Introduction:**

Non-communicable diseases (NCDs) are the leading cause of mortality in the Eastern Mediterranean Region (EMR), driven largely by modifiable behavioral and metabolic risk factors such as unhealthy diet, physical inactivity, tobacco use, and obesity. Innovative and context-sensitive interventions are increasingly recognized as essential to address these challenges, particularly in settings characterized by sociocultural constraints, fragile health systems, and humanitarian crises. This systematic review aimed to examine evidence on innovative approaches for addressing NCD risk factors in the EMR.

**Methods:**

A systematic review was conducted in accordance with PRISMA guidelines. Electronic databases (PubMed/MEDLINE, Scopus, Web of Science, and Embase) were searched for studies published between January 2000 and December 2025. Eligible studies evaluated innovative or context-sensitive interventions targeting modifiable NCD risk factors in EMR populations and included randomized controlled trials, quasi-experimental studies, natural experiments, and field trials. Data were extracted using a standardized form, and methodological quality was assessed using the Mixed Methods Appraisal Tool (MMAT). Due to heterogeneity, findings were synthesized narratively.

**Results:**

Ten studies met the inclusion criteria, with most conducted in Tunisia and additional evidence from Jordan, Palestine, Iran, and Saudi Arabia. Interventions included integrated mental health and NCD programs, community-based initiatives, workplace interventions, culturally adapted health promotion programs, and system-level incentive models. These interventions demonstrated improvements in cardiometabolic outcomes, dietary behaviors, and physical activity, although effects varied across settings. Tobacco-related outcomes were inconsistent, and composite cardiovascular risk scores showed limited improvement. No studies implemented interactive digital health platforms; one study used pedometers as passive monitoring tools.

**Conclusions:**

Innovative, community-based, culturally responsive, and multisectoral interventions show promise in addressing NCD risk factors in the EMR. However, evidence remains limited, geographically concentrated, and methodologically heterogeneous. The absence of evaluated digital health interventions highlights a gap in the current evidence base. Future research should prioritize rigorous designs, scalable models, and evaluation of digital health strategies to strengthen NCD prevention across the region.

**Systematic review registration:**

https://www.crd.york.ac.uk/PROSPERO/view/CRD420251064273.

## Introduction

Noncommunicable diseases (NCDs), including cardiovascular diseases, cancers, respiratory diseases, and diabetes, are the leading causes of mortality worldwide, accounting for approximately 75% of all non-pandemic-related deaths globally (1). In 2021 alone, NCDs were responsible for at least 43 million deaths, with cardiovascular diseases contributing 19 million deaths, cancers 10 million, chronic respiratory diseases 4 million, and diabetes over 2 million. Together, these conditions account for approximately 80% of premature mortality (1).

Although historically associated with high-income countries, the burden of NCDs has shifted disproportionately to low- and middle-income countries (LMICs) (2). This burden is particularly pronounced in the Eastern Mediterranean Region (EMR), where NCDs account for approximately 60–66% of all deaths, reaching up to 89% in some countries, including Lebanon, Tunisia, Bahrain, and Egypt (3). The impact extends beyond adult populations, with increasing prevalence of risk factors among younger age groups; for example, overweight and obesity rates among adolescents aged 13–17 years are estimated at 19.8 and 9.7%, respectively (3).

The high burden of NCDs in the EMR is driven by a combination of structural and behavioral determinants, including limited healthcare infrastructure, delayed diagnosis, restricted access to essential medicines, and widespread exposure to modifiable risk factors such as unhealthy diet, physical inactivity, and tobacco use. These challenges are further exacerbated by regional stressors, including armed conflict, population displacement, climate change, and economic instability, which constrain access to preventive services and influence health behaviors (4–8).

Beyond their health impact, NCDs impose substantial economic and social burdens on individuals, households, and national economies. Premature mortality and disability reduce workforce participation and productivity, while households face high direct and indirect healthcare costs, often leading to financial hardship and deepening inequalities (7, 9–11). At a macroeconomic level, global estimates suggest that productivity losses related to NCDs may exceed US$30 trillion between 2011 and 2030 (7), with similar patterns likely affecting EMR countries.

Despite increasing policy attention, efforts to reduce NCD risk factors in the EMR have yielded mixed results. Previous studies indicate that while community-based and health promotion interventions often achieve high levels of engagement, their impact on sustained behavior change remains limited (12). Moreover, implementation of national NCD strategies varies considerably across countries; for instance, by 2024, only 10 of the 22 EMR countries had incorporated youth mental health into their NCD frameworks, and few had successfully scaled population-level interventions promoting healthy diet and physical activity (3).

In response to these challenges, there is growing interest in innovative and context-sensitive approaches to NCD prevention. Digital health interventions (DHIs), including mobile health platforms, telemedicine, and clinical decision support systems, have shown potential to support behavioral change and improve disease management (13, 14). During the COVID-19 pandemic, several EMR countries rapidly expanded digital health services to maintain continuity of care for patients with chronic conditions (15). Emerging technologies, such as artificial intelligence–assisted monitoring and remote health platforms, further offer opportunities to enhance personalized care and prevention strategies, although implementation challenges persist (16).

At the same time, community-based and multisectoral approaches adapted to local sociocultural contexts have demonstrated promising results. Examples include culturally tailored lifestyle interventions, integration of NCD services within refugee health systems, and policy-level strategies such as taxation of sugar-sweetened beverages and tobacco control measures (17–19). However, structural barriers, including governance limitations, fragmented health systems, and challenges in scaling interventions, continue to constrain their effectiveness and sustainability.

Despite these developments, important evidence gaps remain. Existing literature highlights a limited number of rigorously designed intervention studies in the EMR, substantial heterogeneity in outcomes, and insufficient evaluation of implementation processes and scalability. In particular, there is a lack of comprehensive synthesis of innovative and context-sensitive strategies targeting NCD risk factors in the region.

Therefore, this systematic review aims to synthesize evidence on innovative and context-sensitive interventions addressing NCD risk factors in the EMR. Specifically, the objectives are to:

1. identify digital, community-based, and cross-sectoral strategies implemented in EMR countries;
2. assess their effectiveness in modifying behavioral and clinical outcomes;
3. examine community-based models with demonstrated or potential success; and.
4. document cross-sectoral approaches, including public–private partnerships and regulatory interventions.

The findings of this review aim to inform policy and practice by identifying scalable, culturally appropriate, and context-sensitive strategies to reduce the burden of NCDs in the EMR.

## Methods

### Study design and registration

This study was conducted as a systematic review to synthesize evidence on innovative and context-sensitive approaches addressing modifiable risk factors for non-communicable diseases (NCDs) in the Eastern Mediterranean Region (EMR). The review protocol was prospectively registered in the International Prospective Register of Systematic Reviews (20), PROSPERO (Registration number: CRD420251064273). The review was conducted and reported in accordance with the PRISMA guidelines (2020 statement) (21).

### Eligibility criteria

Eligibility criteria were defined *a priori* using the Population, Intervention, Comparator, Outcomes, and Study Design (PICOS) framework.

### Population

Studies conducted among populations residing in countries classified within the Eastern Mediterranean Region according to the World Health Organization regional classification were eligible. No restrictions were applied regarding age, sex, or specific population groups.

### Interventions

We included studies evaluating innovative and/or context-sensitive interventions targeting modifiable NCD risk factors, including tobacco use, unhealthy diet, physical inactivity, harmful use of alcohol, and obesity and other metabolic risk factors. Innovative approaches were defined using a context-sensitive framework adapted to the realities of the Eastern Mediterranean Region (EMR). In this review, innovation does not necessarily imply global novelty, but rather the introduction, adaptation, or implementation of strategies that are new or substantially improved within a specific local, cultural, or health system context (22, 23). This includes interventions that address contextual constraints such as limited resources, fragile health systems, sociocultural barriers, or population-specific needs (23, 24).

To improve conceptual clarity and consistency, interventions were further classified according to their level of innovation: (1) incremental innovation, referring to adaptations of existing evidence-based approaches to local contexts; (2) adaptive or delivery innovation, involving changes in how interventions are implemented or integrated within systems; and (3) transformative innovation, referring to the introduction of novel technologies or fundamentally new models of care (25, 26).

### Comparator

Studies with or without comparator groups were included. Where applicable, comparators included standard care, no intervention, or alternative interventions.

### Outcomes

#### The primary outcomes included

- Changes in the prevalence or level of NCD risk factors
- Behavioral modifications such as smoking cessation, dietary changes, or increased physical activity

#### Secondary outcomes included

- Clinical indicators such as body mass index (BMI), blood pressure, and glycemic control
- Implementation outcomes, including acceptability, feasibility, and sustainability of interventions.

### Study design

Eligible study designs included randomized controlled trials, quasi-experimental studies, cohort studies, and controlled before–after studies. Qualitative and mixed-methods studies were also included if they evaluated implementation processes or contextual adaptations of interventions. Reviews, editorials, commentaries, and conference abstracts without full data were excluded.

### Information sources and search strategy

A comprehensive literature search was conducted in the following electronic databases: PubMed/MEDLINE, Scopus, Web of Science, and Embase. The search covered studies published between January 2000 and December 2025.

The search strategy combined Medical Subject Headings (MeSH) and free-text terms related to non-communicable diseases, risk factors, innovation, digital health, community-based interventions, and the Eastern Mediterranean Region. Boolean operators (AND/OR) were used to combine search terms appropriately.

In addition, the reference lists of all included studies were manually screened to identify additional relevant publications. Only studies published in English were considered.

### Study selection

All records identified through the database searches were imported into EndNote reference management software, and duplicate records were removed.

**Figure 1:**
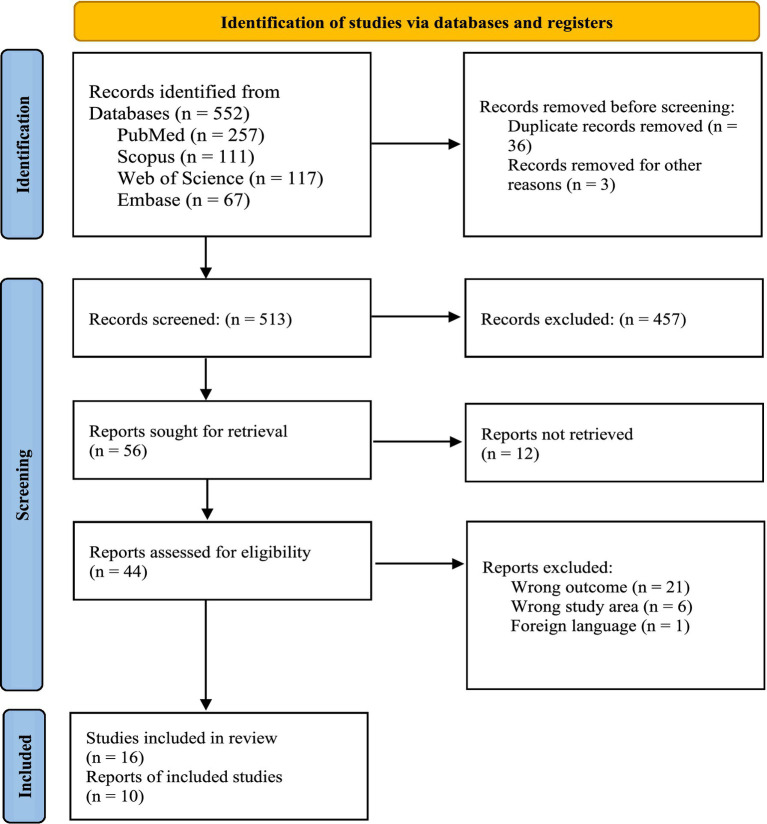
PRISMA flow diagram illustrated study selection process.

Two independent reviewers screened titles and abstracts to determine eligibility. Full texts of potentially relevant studies were subsequently assessed against the predefined inclusion and exclusion criteria. Any disagreements between reviewers were resolved through discussion, and where necessary, consultation with a third reviewer. The study selection process is illustrated using a PRISMA flow diagram (Figure 1).

### Data extraction

A standardized data extraction form was developed and pilot-tested prior to use. From each included study, the following information was extracted: author(s) and year of publication, country of study, study design, target population, description of the intervention, NCD risk factor addressed, duration of the intervention, outcomes measured, key findings, and implementation characteristics. Data extraction was conducted independently by two reviewers to ensure accuracy and consistency, and any discrepancies were resolved through discussion and consensus.

### Quality assessment

The methodological quality and risk of bias of included studies were assessed independently by two reviewers using the Mixed Methods Appraisal Tool (MMAT, version 2018) (27). The MMAT allows the appraisal of qualitative, quantitative, and mixed-methods studies within systematic reviews (Table 1).

**Table 1:** Quality assessment.

Study	Country	Study design	Screening Q1: clear research question?	Screening Q2: data address research question?	Criterion 1	Criterion 2	Criterion 3	Criterion 4	Criterion 5	Overall comments
Powell et al. (28)	Jordan	experiment study design	Yes	Yes	Yes	Yes	Yes	Yes	Yes	100%
Maatoug et al. (29)	Tunisia	Quasi-experimental (pre-post with intervention and control groups)	Yes	Yes	Yes	Yes	Yes	Yes	Yes	100%
Bhiri et al. (32)	Tunisia	Quasi-experimental study (pre- and post-assessments with intervention and control groups).	Yes	Yes	Yes	Yes	Yes	Yes	No	80%
Zammit et al. (30)	Tunisia	quasi-experimental study	Yes	Yes	Yes	Yes	Yes	Yes	Yes	100%
Agbaria et al. (33)	Palestine	First phase consisted of a quasi-experimental study and the second phase included community-wide dissemination	Yes	Yes	Yes	Yes	Yes	Yes	Yes	100%
Sahli et al. (31)	Tunisia	Quasi-experimental	Yes	Yes	Yes	Yes	Yes	Yes	Yes	100%
Asadi-Aliabadi et al. (34)	Iran	a field trial study	Yes	Yes	Yes	Yes	Yes	Yes	Yes	100%
Ghammam et al. (35)	Tunisia	Cross-sectional surveys	Yes	Yes	Yes	Yes	Yes	Yes	No	80%
Aljasir et al. (36)	Saudi Arabia	quasi-experimental study	Yes	Yes	Yes	Yes	Yes	Yes	Yes	100%
Zammit et al. (37)	Tunisia	quasi-experimental study	Yes	Yes	Yes	Yes	Yes	Yes	Yes	100%

Disagreements between reviewers were resolved through discussion. Studies were not excluded based on methodological quality; however, the results of the quality assessment were considered when interpreting the findings.

### Data synthesis

Due to the heterogeneity in study designs, intervention strategies, populations, and outcome measures, a quantitative meta-analysis was not feasible. Therefore, a narrative synthesis was conducted, and findings were organized thematically according to the type of innovative intervention strategy, targeted NCD risk factors, implementation characteristics, and reported effectiveness outcomes.

## Result

### Study selection

The database search identified a total of 552 records. After removing duplicates, 513 records remained for title and abstract screening. Following screening, 44 full-text articles were assessed for eligibility, and 10 studies met the inclusion criteria and were included in the final synthesis.

### Study characteristics

The review included 10 studies conducted across countries in the Eastern Mediterranean Region (Table 2). Most studies were conducted in Tunisia (*n* = 7), followed by Jordan (*n* = 1), Palestine (*n* = 1), and a multi-country context including Iran and Saudi Arabia (*n* = 1) (Table 3).

**Table 2:** Characteristics of included studies: studies examining innovative and context-sensitive approaches for NCD risk factor reduction in the Eastern Mediterranean Region.

Study	Country	Study design	Target population	Sample size	Intervention duration	NCD risk factors targeted	Intervention type	Innovation component
Powell et al. (28)	Jordan	Natural experiment	Syrian refugees & Jordanians	600	18 months	CVD, hypertension, diabetes	Integrated physical + mental health education	Mental health integration with traditional NCD education
Maatoug et al. (29)	Tunisia	Quasi-experimental	Schoolchildren, workers, community adults	~4,000 (schools), ~2,000 (workplaces), ~1,900 (neighborhoods)	3 years	Tobacco, diet, physical inactivity	Multi-sectoral community-based program	Concurrent targeting of schools, workplaces, and neighborhoods
Bhiri et al. (32)	Tunisia	Quasi-experimental	Industrial employees	1,775 (pre), 2,113 (post)	3 years	Poor nutrition, physical inactivity, tobacco	Workplace-based health promotion	Training existing occupational medical staff as intervention deliverers
Zammit et al. (30)	Tunisia	Quasi-experimental	Industrial employees	1,000	3 years	Poor diet, physical inactivity, tobacco	Community engagement program	Open days, health screening, local radio broadcasting
Agbaria et al. (33)	Palestine	Quasi-experimental + dissemination	Palestinian-Arab women	55	6 months	Physical inactivity, poor nutrition	Culturally adapted group lifestyle intervention	Diabetes Prevention Program adaptation for cultural/religious sensitivities
Sahli et al. (31)	Tunisia	Quasi-experimental	Community adults	2,000	3 years	Smoking, physical inactivity, diet	Community-integrated lifestyle intervention	Monthly open sensitization days at supermarkets
Asadi-Aliabadi et al. (34)	Iran	Field trial	Community adults	2,446	5 months	Hypertension, hyperlipidemia, obesity	Multi-component health worker intervention	Performance-based incentives for non-physician health workers
Ghammam et al. (35)	Tunisia	Cross-sectional	Community adults (18–65 years)	920 per group	3 years	Hypertension, physical inactivity, diet	Community-based lifestyle promotion	Neighborhood-level intervention during political transition
Aljasir et al. (36)	Saudi Arabia	Quasi-experimental	Military personnel	267	6 months	Diabetes, hypertension, obesity	Military health promotion program	Comprehensive screening + education in military setting
Zammit et al. (37)	Tunisia	Quasi-experimental	Schoolchildren	204	1 year follow-up	Tobacco, physical inactivity, poor diet	School-based prevention program	Sustainability evaluation post-intervention

CVD, cardiovascular disease; NCD, Non-Communicable Disease.

**Table 3:** Detailed study characteristics and settings.

Study ID	First author (Year)	Specific setting	Age range or mean (years)	Sex distribution (% Male)	Socioeconomic context	Vulnerable groups	Sampling method	Data collection period
1	(28)	Three community health clinics	18–75 (largest: 45–54)	30.1%	76.7% unemployed; 48.5% earn <200 JOD/month	Syrian refugees, low-income Jordanians	Physician referral + clinic flyers	Feb-Apr 2017 (post: 2013–2014)
2	(29)	Schools, workplaces, neighborhoods	Schools: 11–16 (mean 13.2) Workplaces: mean 32.2–38.9 Community: 18–65 (mean 37.2–38.6)	Schools: 50.2% Workplaces: 64.7% Community: 43.2%	Post-revolution Tunisia	Schoolchildren, workers facing urbanization stress	Random sampling	Pre: 2009 Post: 2013–2014
3	(32)	Six industrial companies	Intervention: 32.25 ± 8.11 Control: 35.40 ± 8.75	64.7% (intervention)	Low/medium socioeconomic level	Industrial workers	Convenience (matched companies)	Pre: 2009–2010 Post: 2013–2014
4	(30)	Six companies	48.4 ± 12.7 (intervention)	65.5% (intervention post)	Low (55.2%), Medium (34.0%), High (10.8%)	Industrial employees	Cluster sampling	2009–2014
5	(33)	Two community centers, East Jerusalem	Intervention: 37.20 ± 13.22 Comparison: 57 ± 11.3	0% (all female)	Not specified	Palestinian-Arab women	Convenience sample	Jun-Dec 2016
6	(31)	Neighborhoods, workplaces, schools, primary care	Control: 38.61 ± 13.73 to 40.43 ± 13.96	Control: 28.8% (pre) to 34.3% (post)	Not specified	Community adults	Random selection	2010–2013
7	(34)	32 community health centers, 4 districts	49.27 ± 0.33 (survey 1) 49.38 ± 0.32 (survey 2)	49.9% (survey 1) 49.6% (survey 2)	Not specified	Community adults	Random sampling	Phase 1: Jun-Sep 2018 Phase 2: Sep-Nov 2019
8	(35)	Community neighborhoods	Intervention: 37.20 ± 13.22 to 39.25 ± 13.61 Comparison: 38.61 ± 13.73 to 40.43 ± 13.96	Intervention: 43.2% → 44.2% Comparison: 28.8% → 34.3%	Educational level, employment, socioeconomic level specified	Community adults	Survey methodology	2009–2010 (baseline) 2013–2014 (follow-up)
9	(36)	Military camp, Jeddah	35.8 ± 6.6	100%	94% low military rank; 55% secondary education	Military personnel	Systematic random sample	Not indicated
10	(37)	Schools	Exposed: 12.39 ± 0.68 Not exposed: 12.4 ± 0.72	Exposed: 43.8% Not exposed: 46.5%	Students	Schoolchildren	Random selection	2013–2014 to 2014–2015

JOD, Jordanian Dinar.

Study designs included quasi-experimental studies (*n* = 8), one natural experiment, and one field trial. Sample sizes ranged from 55 to 4,000 participants, and intervention durations ranged from 6 months to 3 years. Target populations were diverse and included refugees, military personnel, school-aged children, community-dwelling adults, and industrial employees.

### Types of innovative strategies addressing NCD risk factors

#### Integrated health–mental health interventions

One study (28) conducted in Jordan evaluated the integration of mental health support within NCD prevention programs among Syrian refugees and low-income Jordanians. The “Healthy Community Clinic Plus Mental Health” model combined traditional NCD education with psychoeducation and solution-focused counseling. The intervention demonstrated significant reductions in cardiovascular risk factors compared with education-only programs, indicating the added benefit of addressing psychological determinants of health (Tables 4, 5).

**Table 4:** Summary of key outcomes by intervention approach.

Intervention approach	Representative studies	Primary clinical/biomarker outcomes	Behavioral outcomes	Implementation outcomes	Direction of effect
Integrated health-mental health model	(28)	BMI: −3.98 (95% CI: −4.16 to −3.80) SBP: −14.49 mmHg (95% CI: −17.96 to −11.02) DBP: −11.41 mmHg FBG: −20.32 mg/dL (95% CI: −28.87 to −11.77) HbA1c: −0.43%	Sustained improvements at 18-month follow-up	Acceptability: High Feasibility: High Reach: High Sustainability: Yes	Positive (synergistic effect)
Multi-sectoral community-based programming	(29, 31, 35)	Hypertension prevalence: ↓4.4% (35.8% → 31.4%) (*p* = 0.006) SBP: *β* = −0.4 (95% CI: −0.76 to −0.06) DBP: *β* = −0.22 (95% CI: −0.38 to −0.07) OR for hypertension: 0.73 (95% CI: 0.59–0.91)	Fruit/vegetable intake (5 servings/day): Schools: 30% → 33.2% (*p* = 0.027) Workplaces: 47.5% → 52.1% (*p* = 0.04) Neighborhoods: 39.4% → 58.4% (*p* < 0.001) Tobacco use (men): ↓7.3% (*p* = 0.03)	Acceptability: High Feasibility: High Reach: Very High (4,000+) Scalability: Yes Sustainability: Variable	Positive (setting-dependent)
Workplace capacity-building	(30, 32)	Not directly reported	Fruit/vegetable intake: 47.5% → 52.1% (*p* = 0.04) Physical activity (recommended): 28.3% → 37.9% (*p* < 0.001) Tobacco use: 39.2% → 37.5% (*p* = 0.43, NS)	Acceptability: High Feasibility: High Reach: Moderate Scalability: Yes	Mixed (positive for diet/activity; neutral for tobacco)
Culturally adapted group interventions	(33)	Weight loss: −2.21 kg (*p* < 0.01)	Vegetable intake: 1.25 → 2.01 servings (*p* < 0.05) Fruit/whole grain intake: 0.73 → 1.92 servings (*p* < 0.05) Physical activity (recommended): 15.1% → 40.1% (*p* < 0.001)	Acceptability: Moderate Feasibility: Moderate Reach: Moderate Scalability: Moderate	Positive
Performance-based incentives for health workers	(34)	Not directly reported	Average daily steps: 4,456 → 64,040 (*p* < 0.001)	Not reported	Positive
Military workplace model	(36)	BMI: −0.4 ± 1.5 kg/m^2^ Waist circumference: −0.9 ± 6.2 cm FBG: −12.3 ± 29.6 mg/dL Total cholesterol: −15.4 ± 40.2 mg/dL	Framingham risk score: −0.1 ± 2.1 points (NS) Diabetes risk score: −0.01 ± 2.5 (NS)	Acceptability: 96% satisfied Feasibility: High Reach: Moderate Scalability: Yes	Mixed (improved individual factors but not composite risk scores)

BMI, Body Mass Index; SBP, Systolic Blood Pressure; DBP, Diastolic Blood Pressure; FBG, Fasting Blood Glucose; HbA1c, Glycated Hemoglobin; CI, Confidence Interval; OR, Odds Ratio; NS, Not Significant.

**Table 5:** Intervention components and delivery characteristics.

Study ID	First author (Year)	Intervention name	Intervention components	Delivery mode	Intervention level	Frequency/intensity	Context-sensitive features
1	(28)	Healthy Community Clinic (HCC) and HCC plus Mental Health (HCC-MH)	NCD education + psychoeducation + solution-focused techniques	Group work model	Primary health care	20 sessions (45 min each), twice monthly for 12 months	Culturally relevant mental health materials for Syrian refugees and Jordanians
2	(29)	“Together in Health” project	Teacher-led sessions, sports tournaments, educative films, smoking cessation, radio sessions, flyers	Mixed modes (teacher-led, media, events)	Community/population	3-year continuous program	Adapted from North Karelia model to Tunisian post-revolution context
3	(32)	3-Year Workplace-Based Intervention Program	Educative films, workshops, open sensitization days, posters, free PA sessions, smoking cessation	On-site workplace delivery	Workplace	3 years	Smoking banned at workplaces; adapted to local sociopolitical climate
4	(30)	“Together in Health”	Open days, health screening, educational broadcasting, primary care physician training	Periodic open days, radio	Community	3 years	Local radio adaptation
5	(33)	Community-Based Lifestyle Intervention (CBLI)	Mediterranean diet, PA, cardiovascular health literacy, behavioral self-regulation, conscious eating	Interactive lectures + 1 h aerobic exercise	Community	6 months	Religious sensitivities considered; culturally adapted DPP
6	(31)	Community-Integrated Lifestyle Intervention Program	Education sessions, open sensitization days on smoking, PA, diet	Monthly Sunday sessions at supermarkets, mass media	Community and individual	Monthly for 3 years	Data collection tools translated and adapted to local language
7	(34)	Not specified	Goal-setting, evidence-based education, operational planning, performance-based incentives	Health worker-delivered	Community	Phase 1: Jun-Sep 2018	Implemented across 32 community health centers
						Phase 2: Sep-Nov 2019	
8	(35)	Not specified	PA promotion, healthy eating, tobacco control education	Multi-approach	Community	3 years	Implemented during political transition
9	(36)	National Guard Health Promotion Program	Health screening, education, risk factor modification	Multi-approach	Community (military)	6 months	Tailored for military personnel
10	(37)	School-based prevention program	Health education, lifestyle promotion	School-based	School	3 years (with 1-year follow-up)	School-based delivery

NCD, Non-Communicable Disease; PA, Physical Activity; DPP, Diabetes Prevention Program.

#### Multi-sectoral community-based programs

Several studies conducted in Tunisia (29–31) (Table 6), evaluated the “Together in Health” program, a multi-sectoral intervention targeting schools, workplaces, and neighborhoods simultaneously. The program was modeled after the North Karelia project and adapted to the Tunisian sociopolitical context (Table 5). This approach reinforced health promotion messages across multiple community settings and leveraged existing institutional structures (Tables 4, 7).

**Table 6:** Summary of innovative strategies identified.

Innovation category	Specific innovation	Study	Country	Key feature
Service integration	Mental health + physical health NCD education	(28)	Jordan	Psychoeducation and solution-focused techniques integrated with traditional NCD education
Multi-sectoral approach	Concurrent targeting of schools, workplaces, and neighborhoods	(29, 30, 31, 35, 37)	Tunisia	Modeled after North Karelia project; adapted to Tunisian sociopolitical context
Workforce development	Training existing occupational medical staff	(32)	Tunisia	Non-external experts ensured routine health promotion and sustainability
Cultural adaptation	DPP adaptation for Palestinian-Arab women	(33)	Palestine	Religious sensitivities, family dynamics, and cultural environment considered
Health system financing	Performance-based incentives for health workers	(34)	Iran	Goal-setting, operational planning, and incentives for non-physician health workers
Setting-based innovation	Monthly open sensitization days at supermarkets	(31)	Tunisia	Commercial entity collaboration for community reach
Technology-enhanced measurement	Validated pedometers for outcome measurement	(33, 34)	Palestine, Iran	Objective physical activity measurement
Mass media utilization	Radio broadcasting for health promotion	(29, 30)	Tunisia	Leveraged existing communication infrastructure

DPP, Diabetes Prevention Program.

**Table 7:** Cross-sector collaboration examples and outcomes.

Collaboration type	Study	Country	Sectors involved	Collaboration description	Key outcomes
Health-education sector	(29, 37)	Tunisia	Health + Education	Teachers delivered health education sessions in schools	~4,000 learners reached; significant dietary improvements; sustainability required curriculum integration
Health-workplace sector	(30, 32)	Tunisia	Public Health + Private Industrial Enterprises	Occupational physicians/nurses trained to deliver NCD prevention	6 companies participated; improved physical activity and diet; tobacco reduction non-significant (p = 0.43)
Health-media sector	(29, 30, 31)	Tunisia	Health + Mass Media (Radio/Newspaper)	Mainstream media coverage; monthly open sensitization days at supermarkets	Increased message reach; reinforced community-level messages; supermarket partnership innovative
Multi-stakeholder primary care	(28)	Jordan	Ministry of Health + Community Clinics + Academic Researchers	Physician referral system; community clinics as intervention sites	600 marginalized participants recruited (refugees + low-income Jordanians); high retention
Health-NGO-community	(33)	Palestine	Academic Institutions + Community Centers + Local Community	Community input for cultural adaptation; centers as intervention sites	High intervention acceptability; significant behavior change in women
Cross-government health system	(34)	Iran	Multiple Community Health Centers + Health System Management	32 centers across 4 districts; performance-based incentives	Multi-site collaboration feasible at scale; improved health system management

#### Workplace capacity-building interventions

One study (32) implemented workplace-based interventions that trained occupational health providers to deliver NCD prevention education and smoking cessation services. By integrating prevention activities into routine occupational health services, the program enhanced sustainability and minimized reliance on external experts (Tables 6, 8).

**Table 8:** Implementation outcomes by study.

Study ID	First author (Year)	Acceptability	Feasibility	Reach	Scalability	Sustainability	Evidence for implementation outcomes
1	(28)	Yes	Yes	Yes	No	Yes	High retention rates; 600 participants completed study; sustained effects at 18 months
2	(29)	Yes	Yes	Yes	Yes	Yes	Reached ~4,000 schoolchildren, ~2,000 workers, ~1,900 community adults; government scalability recommended
3	(32)	Yes	Yes	Yes	Yes	Yes	1,775–2,113 employees reached; occupational staff trained as intervention deliverers enabling sustainability
4	(30)	High	High	High	High	Moderate	1,000 participants; tobacco use reduced in men; recommended supportive environment for sustainability
5	(33)	Moderate	Moderate	Moderate	Moderate	Moderate	55 participants; culturally adapted program; lengthy adaptation process noted
6	(31)	High	Moderate	Moderate	High	Moderate	2,000 participants; demonstrated feasibility in developing country context
7	(34)	Not reported	Not reported	Not reported	Not reported	Not reported	2,446 participants across 32 centers; multi-site collaboration feasible at scale
8	(35)	Yes	Not reported	Yes	Yes	Yes	920 per group; protective effects on BP; scalable to other similar settings
9	(36)	Yes (96% satisfied)	Not reported	Yes	Yes	Yes	267 participants; high satisfaction; effective for specific risk factors
10	(37)	Not reported	Not reported	Yes	Yes	Yes (limited)	204 schoolchildren; limited sustainable effects at 1 year; continuous reinforcement needed

#### Culturally adapted community programs

A culturally adapted diabetes prevention program targeting Palestinian-Arab women in East Jerusalem incorporated Mediterranean dietary guidance (33), culturally appropriate physical activities, and behavioral self-regulation strategies. The intervention also considered religious practices and family dynamics, which improved participant engagement and program acceptability (Tables 5, 6).

#### System-level workforce incentive programs

In Iran (34), an intervention incorporating performance-based incentives for non-physician health workers aimed to improve implementation of NCD prevention programs within primary healthcare settings. The intervention package included goal setting, operational planning, and evidence-based health education (Tables 4, 6).

#### Digital and media-based tools

Limited use of digital technologies was identified in the included studies. One study used pedometers as monitoring tools to assess physical activity levels (34), demonstrating significant increases in daily step counts. However, the pedometer served primarily as a measurement tool rather than a central intervention component (Tables 4, 9).

**Table 9:** Primary and secondary outcomes with effect sizes.

Study ID	First author (Year)	Primary outcomes	Effect size (95% CI)	*p*-value	Secondary outcomes	Effect size (95% CI)	*p*-value	Measurement tools
1	(28)	BMI	−3.98 (−4.16 to −3.80)	<0.001	FBG	−20.32 (−28.87 to −11.77)	<0.001	Anthropometric tools, BP cuff, blood tests
		SBP (mmHg)	−14.49 (−17.96 to −11.02)	<0.001	HbA1c	−0.43%	<0.001	--
		DBP (mmHg)	−11.41	<0.001	LDL/HDL/Triglycerides	Not reported	<0.001	--
2	(29)	Tobacco use (neighborhood)	26.2% → 23.2%	Not reported	Hypertension (workplace)	16.2% → 12.8%	0.027	Standardized questionnaires, biometric measures
		Fruit/vegetable intake (school)	30% → 33.2%	0.027	--	--	---	--
		Fruit/vegetable intake (neighborhood)	39.4% → 58.4%	<0.001	--	--	--	--
3	(32)	Fruit/vegetable intake (≥5 servings)	47.5% → 52.1%	0.04	Subgroup: Office staff dietary improvement	--	0.04	Pretested questionnaire via interview
		Physical activity (recommended)	28.3% → 37.9%	<0.001	Subgroup: >40 years PA	--	<0.001	--
		Tobacco use	39.2% → 37.5%	0.43 (NS)	Subgroup: Workers PA	--	<0.001	--
4	(30)	Tobacco use (men)	↓7.3%	0.03	Physical activity (recommended)	15.1% → 40.1%	<0.001	Questionnaires
		Fruit/vegetable intake (5 servings)	Increased	Not reported	--	--	--	--
5	(33)	Vegetable intake (servings)	1.25 → 2.01	<0.05	Weight loss (kg)	−2.21	<0.01	ADS, questionnaires
		Fruit/whole grain intake (servings)	0.73 → 1.92	<0.05	ADS	4,456 → 64,040	<0.001	--
		Physical activity (recommended)	15.1% → 40.1%	<0.001	--	--	--	--
6	(31)	Hypertension prevalence	35.8% → 31.4% (AOR: 0.73)	0.006	Fruit/vegetable intake (5 servings)	39.4% → 58.4%	<0.001	IPAQ, electronic sphygmomanometer
7	(34)	ADS	4,456 → 64,040	<0.001	Obesity/overweight prevalence	OR: 0.57 (0.34–0.95)	Not reported	Validated pedometer, blood tests, questionnaire
8	(35)	Hypertension prevalence	↓4.4% (35.8% → 31.4%)	0.044	SBP effect	*β* = −0.4 (−0.76 to −0.06)	0.035	Arabic questionnaire, IPAQ, OMRON sphygmomanometer
		SBP (mmHg)	132.4 → 130.6	0.035	DBP effect	*β* = −0.22 (−0.38 to −0.07)	<0.001	--
		DBP (mmHg)	78.7 → 76.9	<0.001	Vegetable intake	1.76 → 2.32 servings	Not reported	--
9	(36)	BMI (kg/m^2^)	−0.4 ± 1.5	<0.005	Framingham risk score	−0.1 ± 2.1	NS	Framingham risk scores, self-administered questionnaire
		Waist circumference (cm)	−0.9 ± 6.2	<0.005	Diabetes risk score	−0.01 ± 2.5	NS	
		FBG (mg/dL)	−12.3 ± 29.6	<0.005	Program satisfaction	96%	N/A	
		Total cholesterol (mg/dL)	−15.4 ± 40.2	<0.005				
10	(37)	Tobacco experimentation (exposed)	↑2.9%	0.77 (NS)	Physical activity	No significant change	NS	Biometric measurements, questionnaires
		Tobacco experimentation (non-exposed)	↑11.1%	0.001	Fast food/fried food consumption	No significant change	NS	

BMI, Body Mass Index; SBP, Systolic Blood Pressure; DBP, Diastolic Blood Pressure; FBG, Fasting Blood Glucose; HbA1c, Glycated Hemoglobin; PA, Physical Activity; ADS, Average Daily Steps; IPAQ, International Physical Activity Questionnaire; AOR, Adjusted Odds Ratio; OR, Odds Ratio; CI, Confidence Interval; NS, Not Significant; N/A, Not Applicable.

Mass media approaches were used in several Tunisian interventions (29, 30), including radio campaigns and public awareness activities. While these approaches improved population-level reach, they lacked interactive features commonly associated with modern digital health technologies (Tables 5, 6).

Notably, none of the included studies utilized smartphone applications, mobile messaging services, telemedicine platforms, or web-based interventions for NCD risk factor modification.

### Effectiveness of community-based intervention models

#### Tunisian multi-sectoral model

Community-based interventions in Tunisia demonstrated improvements across multiple NCD risk factors (Table 4). In neighborhood settings, fruit and vegetable consumption increased significantly from 39.4 to 58.4%, while hypertension prevalence declined from 35.8 to 31.4%. Improvements were also observed in systolic and diastolic blood pressure (31).

Workplace interventions reported increased adherence to dietary recommendations and physical activity guidelines (32). For example, daily fruit and vegetable consumption increased from 47.5 to 52.1%, while physical activity participation increased from 28.3 to 37.9%.

School-based interventions (30), also demonstrated improvements in dietary behaviors among students; however, follow-up evaluations suggested that sustained behavioral change required ongoing reinforcement (Table 9).

#### Clinical and behavioral outcomes

##### Cardiovascular and metabolic outcomes

Several studies reported improvements in cardiovascular and metabolic risk indicators (28, 31, 35). These included reductions in blood pressure, fasting blood glucose, cholesterol levels, and HbA1c. For example, integrated interventions in Jordan demonstrated sustained reductions in BMI, systolic and diastolic blood pressure, and glycemic indicators over an 18-month follow-up period (Table 9).

##### Anthropometric outcomes

Anthropometric improvements were also reported (36). A workplace intervention among military personnel in Saudi Arabia showed modest reductions in BMI and waist circumference, although changes in overall cardiovascular risk scores were not statistically significant (Table 4).

##### Behavioral risk factors

Dietary behaviors improved through several interventions, particularly in multi-setting community programs (29, 30, 32, 33, 37). Physical activity levels also increased substantially in culturally adapted and workplace-based programs.

Tobacco use outcomes were mixed. Some interventions reported significant reductions in tobacco consumption (30), while others observed non-significant changes (32).

##### Implementation outcomes

Across the included studies, several implementation outcomes were reported (29–33, 36, 37). Acceptability of interventions was generally high, with one workplace intervention reporting participant satisfaction levels as high as 96%.

Feasibility was rated as high in seven studies and moderate in three studies. Multi-sectoral programs demonstrated particularly strong reach, with some interventions engaging up to 4,000 participants.

Sustainability outcomes varied. Integrated health–mental health models demonstrated continued improvements over a medium-term follow-up period of up to 18 months, although these effects were observed during ongoing intervention delivery rather than after withdrawal. School-based interventions showed limited maintenance of behavioral change over follow-up periods, particularly where ongoing reinforcement was not sustained. (Table 8).

To improve comparability across studies with heterogeneous outcome measures, a standardized summary of intervention effects was developed (Table 10). Across the included studies, most interventions demonstrated positive effects on both behavioral and cardiometabolic outcomes.

**Table 10:** Standardized summary of intervention effects across included studies.

Study ID	Outcome domain	Outcome	Effect	*p*-value	Direction	Magnitude	Interpretation
Powell et al. (28)	Cardiometabolic	BMI	−3.98	<0.001	Positive	Large	Clinically meaningful reduction
	Cardiometabolic	SBP	−14.49 mmHg	<0.001	Positive	Large	Strong BP reduction
	Cardiometabolic	DBP	−11.41 mmHg	<0.001	Positive	Large	Strong BP reduction
	Cardiometabolic	FBG	−20.32 mg/dL	<0.001	Positive	Moderate	Improved glycemic control
	Cardiometabolic	HbA1c	−0.43%	<0.001	Positive	Moderate	Clinically relevant improvement
Maatoug et al. (29)	Behavioral	Tobacco use	26.2% → 23.2%	NR	Positive	Small	Slight reduction
	Behavioral	Fruit/vegetable intake (school)	30% → 33.2%	0.027	Positive	Small	Modest improvement
	Behavioral	Fruit/vegetable intake (community)	39.4% → 58.4%	<0.001	Positive	Large	Strong dietary improvement
	Cardiometabolic	Hypertension	16.2% → 12.8%	0.027	Positive	Moderate	Meaningful reduction
Bhiri et al. (32)	Behavioral	Fruit/vegetable intake	47.5% → 52.1%	0.04	Positive	Small	Modest improvement
	Behavioral	Physical activity	28.3% → 37.9%	<0.001	Positive	Moderate	Meaningful increase
	Behavioral	Tobacco use	39.2% → 37.5%	0.43	Neutral	None	No significant effect
Zammit et al. (30)	Behavioral	Tobacco use (men)	↓7.3%	0.03	Positive	Moderate	Reduction in smoking
	Behavioral	Physical activity	15.1% → 40.1%	<0.001	Positive	Large	Strong increase
	Behavioral	Diet	Increased	NR	Positive	Moderate	Improved dietary behavior
Agbaria et al. (33)	Behavioral	Vegetable intake	1.25 → 2.01	<0.05	Positive	Moderate	Improved diet
	Behavioral	Fruit/whole grains	0.73 → 1.92	<0.05	Positive	Moderate	Improved diet
	Behavioral	Physical activity	15.1% → 40.1%	<0.001	Positive	Large	Strong increase
	Anthropometric	Weight	−2.21 kg	<0.01	Positive	Moderate	Meaningful weight loss
	Behavioral	Steps (ADS)	4,456 → 64,040	<0.001	Positive	Large	Substantial increase
Sahli et al. (31)	Cardiometabolic	Hypertension	35.8% → 31.4%	0.006	Positive	Moderate	Population-level improvement
	Behavioral	Diet	39.4% → 58.4%	<0.001	Positive	Large	Strong dietary change
Asadi-Aliabadi et al. (34)	Behavioral	Steps (ADS)	4,456 → 64,040	<0.001	Positive	Large	Substantial increase
	Cardiometabolic	Obesity	OR 0.57	NR	Positive	Moderate	Reduced risk
Ghammam et al. (35)	Cardiometabolic	Hypertension	↓4.4%	0.044	Positive	Small–Moderate	Modest reduction
	Cardiometabolic	SBP	−1.8 mmHg	0.035	Positive	Small	Limited clinical effect
	Cardiometabolic	DBP	−1.8 mmHg	<0.001	Positive	Small	Limited effect
	Behavioral	Diet	1.76 → 2.32 servings	NR	Positive	Moderate	Improved intake
Aljasir et al. (36)	Anthropometric	BMI	−0.4	<0.005	Positive	Small	Limited reduction
	Anthropometric	Waist circumference	−0.9 cm	<0.005	Positive	Small	Minor improvement
	Cardiometabolic	FBG	−12.3 mg/dL	<0.005	Positive	Moderate	Improved glucose
	Cardiometabolic	Cholesterol	−15.4 mg/dL	<0.005	Positive	Moderate	Clinically relevant
	Composite risk	Risk scores	NS	NS	Neutral	None	No improvement
Zammit et al. (37)	Behavioral	Tobacco (exposed)	↑2.9%	0.77	Neutral	None	No significant change
	Behavioral	Tobacco (non-exposed)	↑11.1%	0.001	Negative	Moderate	Significant worsening
	Behavioral	Physical activity	NS	NS	Neutral	None	No effect
	Behavioral	Diet	NS	NS	Neutral	None	No effect

NR, Not reported; NS, Not significant; SBP, systolic blood pressure; DBP, diastolic blood pressure; FBG, fasting blood glucose; ADS, average daily steps.

In particular, dietary behaviors and physical activity showed the most consistent improvements, with several studies reporting moderate to large increases in fruit and vegetable intake and substantial gains in physical activity levels. Cardiometabolic outcomes, including blood pressure, glycemic control, and anthropometric measures, also improved in most studies, although the magnitude of effects varied from small to large depending on the intervention type and population.

In contrast, tobacco-related outcomes were mixed, with some studies reporting modest reductions, while others showed no significant change or even worsening trends in specific subgroups. Interventions targeting composite cardiovascular risk scores demonstrated limited or non-significant effects, suggesting that changes in individual risk factors may not always translate into measurable improvements in overall risk profiles.

Overall, the standardized synthesis indicates that while most interventions produced beneficial effects, the magnitude and consistency of outcomes varied across domains, highlighting the need for more uniform outcome reporting and robust evaluation methods in future studies.

## Discussion

This systematic review synthesized available evidence on innovative approaches for addressing non-communicable disease (NCD) risk factors in the Eastern Mediterranean Region (EMR). The findings indicate that community-based, culturally adapted, and multisectoral interventions can produce measurable improvements in behavioral and clinical risk factors. However, the current evidence base remains geographically concentrated and methodologically heterogeneous, highlighting persistent gaps in the implementation and evaluation of innovative prevention strategies across the region.

One of the most important findings of this review is the effectiveness of integrated health–mental health interventions in improving cardiometabolic outcomes among vulnerable populations. The intervention conducted in Jordan demonstrated significant reductions in body mass index (BMI), blood pressure, and glycemic markers when mental health support was integrated into NCD education programs. These results align with previous research demonstrating the strong bidirectional relationship between psychological distress and cardiometabolic disease risk, particularly in populations exposed to conflict, displacement, and socioeconomic instability (4, 5, 38). Evidence from intervention research among Syrian refugees in Jordan similarly indicates that combining mental health awareness with NCD prevention programs can significantly reduce cardiovascular risk factors and improve metabolic indicators over time (28). This integrated model may enhance adherence to lifestyle modifications and treatment recommendations by addressing psychosocial barriers such as stress, trauma, and limited coping mechanisms. In fragile settings where populations face multiple health and social vulnerabilities, integrated models that simultaneously address mental and physical health determinants may therefore represent a particularly effective strategy for NCD prevention (12).

Another key observation from this review is that many successful interventions relied on multisectoral collaboration involving health systems, educational institutions, workplaces, community organizations, and civil society actors. Multisectoral community-based interventions, particularly those implemented in Tunisia, demonstrated improvements in dietary behaviors, physical activity, and hypertension prevalence. These findings are consistent with global policy frameworks that emphasize multisectoral action as a cornerstone of effective NCD prevention (8). Addressing the upstream determinants of NCD risk factors, including food environments, urban design, and occupational settings—requires coordinated action across sectors beyond healthcare alone. Nevertheless, the sustainability of such approaches in the EMR remains uncertain. Regional assessments have highlighted structural challenges including limited financing mechanisms, fragmented governance systems, and weak regulatory frameworks for public–private partnerships, all of which may constrain the long-term institutionalization and scalability of multisectoral interventions (18). Strengthening governance structures and policy coordination may therefore be critical for translating successful pilot programs into sustainable population-level strategies.

Culturally adapted interventions also emerged as an important element of effective NCD prevention programs in the region. The culturally tailored diabetes prevention intervention targeting Palestinian Arab women incorporated dietary guidance aligned with Mediterranean dietary patterns, culturally appropriate physical activities, and behavioral self-regulation strategies that accounted for religious practices and family dynamics. These adaptations appeared to improve participant engagement and acceptability. In many EMR countries, sociocultural norms strongly shape health behaviors, particularly among women who may face structural barriers to physical activity or healthcare access. Previous regional analyses have similarly emphasized that culturally responsive health promotion programs are essential for achieving sustainable behavioral change in settings where social expectations, gender roles, and religious practices influence health behaviors (3). Consequently, interventions that incorporate culturally sensitive messaging and community engagement strategies may be more effective than standardized programs developed in different sociocultural contexts.

Institution-based interventions, including workplace and military-based programs, demonstrated improvements in certain behavioral indicators such as dietary practices and physical activity levels. However, their impact on tobacco cessation and composite cardiovascular risk scores was inconsistent. This finding is consistent with broader global evidence indicating that institutional environments can support short-term behavioral modifications but may have limited effectiveness in addressing deeply entrenched risk behaviors such as tobacco use without complementary policy-level interventions. Structural measures such as tobacco taxation, marketing restrictions, smoke-free policies, and regulation of unhealthy food environments have been shown to produce more substantial population-level reductions in NCD risk factors (18, 19). Therefore, institution-based interventions may be most effective when integrated within broader policy frameworks that address both individual behaviors and structural determinants of health.

An important observation from this review is the absence of evaluated interactive digital health interventions targeting NCD risk factors within the included studies. However, this finding should be interpreted with caution. Rather than indicating a lack of implementation, it more likely reflects a limited body of published and rigorously evaluated digital NCD prevention interventions in the EMR. Despite increasing global evidence demonstrating the effectiveness of mobile health applications, telehealth services, and digital self-management platforms in supporting behavioral change and chronic disease management (13, 14), few such interventions have been formally assessed and reported within the regional literature.

This gap is notable in the context of the rapid expansion of digital health infrastructure across several EMR countries during the COVID-19 pandemic, when teleconsultation services and remote health platforms were implemented to maintain continuity of care for individuals with chronic diseases (15). However, these initiatives have primarily focused on clinical service delivery and may not yet be reflected in peer-reviewed studies evaluating preventive outcomes. Therefore, the absence of digital approaches in the interventions identified in this review should be understood as a gap in the current evidence base rather than a confirmed absence of implementation (16).

Several structural and contextual factors may contribute to this evidence gap. These include variability in digital infrastructure, limited integration of digital tools within primary healthcare systems, regulatory and governance challenges, and disparities in digital literacy and access across populations in the region. Furthermore, many digital health initiatives in the EMR have focused primarily on treatment and disease management rather than prevention or population-level health promotion strategies. Emerging research suggests that digital technologies, including mobile health platforms, artificial intelligence–assisted monitoring systems, and remote behavioral coaching tools, have the potential to enhance chronic disease prevention by improving patient engagement, monitoring health behaviors, and supporting personalized interventions (39). Accordingly, there is a clear need for future primary research to design, implement, and rigorously evaluate digital health strategies for NCD prevention in the EMR, particularly those that are scalable, culturally appropriate, and integrated within existing health systems.

Finally, the findings of this review underscore the need for stronger evidence on the effectiveness and scalability of innovative NCD prevention strategies in the EMR. Most included studies employed quasi-experimental designs and were conducted in a limited number of countries, particularly Tunisia, which limits the generalizability of the findings. Expanding the evidence base will require rigorously designed randomized or controlled intervention studies conducted across diverse sociopolitical and health system contexts within the region. In addition, implementation science approaches should be incorporated to evaluate scalability, sustainability, and cost-effectiveness of interventions.

The interpretation of these findings should be considered in light of the methodological quality of the included studies. According to the MMAT assessment, most studies were rated as moderate to high quality, although the majority employed quasi-experimental designs, which may be subject to selection bias and limited causal inference.

Notably, some of the strongest and most consistent effects, particularly those observed in integrated health–mental health interventions, were supported by higher-quality evidence, including the natural experiment conducted in Jordan. Similarly, several community-based interventions in Tunisia demonstrated high methodological quality and consistent positive outcomes, particularly for dietary behaviors and physical activity.

However, other findings, including mixed results for tobacco use and limited effects on composite cardiovascular risk scores, were often derived from studies with weaker designs or less robust reporting. This variability suggests that some observed effects may be influenced by study design limitations rather than true intervention effectiveness.

From a policy perspective, the findings suggest that reducing the growing burden of NCDs in the EMR will require comprehensive prevention strategies that combine community-based engagement, culturally responsive health promotion programs, integrated mental and physical health services, and supportive multisectoral governance frameworks. Strengthening digital health integration and investing in scalable intervention models may further enhance the effectiveness and reach of NCD prevention efforts across the region.

## Limitations

Several limitations should be considered when interpreting the findings of this review. First, the number of eligible studies was relatively small and geographically concentrated, with the majority conducted in Tunisia. This geographic imbalance substantially limits the representativeness and generalizability of the findings across the diverse countries of the Eastern Mediterranean Region (EMR), where health systems, sociocultural contexts, and policy environments differ considerably. Second, most included studies employed quasi-experimental designs, and only a limited number used rigorous experimental methodologies, which may increase the risk of bias and reduce the strength of causal inference. Third, heterogeneity in intervention designs, populations, outcome measures, and follow-up periods precluded quantitative meta-analysis and necessitated a narrative synthesis approach.

Fourth, the review included only studies published in English, which introduces potential language bias and may have resulted in the exclusion of relevant research published in French, Arabic, or Persian. This is particularly important in the EMR context, where a substantial body of research from Francophone North African countries (e.g., Morocco, Algeria, Tunisia) and Iran may be published in non-English languages. The English-only restriction was applied for feasibility reasons and to ensure consistency in screening, data extraction, and quality appraisal; however, it should be acknowledged as a significant limitation. Finally, variations in reporting implementation outcomes across studies limited the ability to systematically compare scalability and sustainability of interventions. Future reviews should incorporate multilingual search strategies, including regional databases and grey literature sources, to provide a more comprehensive and representative evidence base.

## Implications for future research

Future research should prioritize the development and evaluation of rigorously designed interventions across a broader range of EMR countries to improve the generalizability of evidence on NCD prevention strategies. There is a particular need for randomized or controlled intervention studies examining the effectiveness of integrated mental and physical health models, multisectoral prevention programs, and culturally tailored interventions in diverse sociocultural contexts. In addition, future studies should explore the potential of digital health innovations, including mobile applications, telehealth platforms, and AI-assisted monitoring, to enhance prevention and self-management of NCD risk factors. Implementation research is also needed to better understand how multisectoral collaborations can be sustained, scaled, and embedded within existing health systems and policy frameworks. Strengthening evaluation frameworks and incorporating standardized implementation indicators may further support the translation of successful pilot interventions into scalable regional strategies.

## Conclusion

Community-based, culturally adapted, and integrated interventions can produce meaningful improvements in key NCD risk factors in the Eastern Mediterranean Region. However, the absence of evaluated digital health strategies and the limited availability of rigorous implementation research highlight important gaps in the current evidence base. Strengthening multisectoral collaboration, expanding culturally responsive programs, and integrating digital innovations into prevention strategies will be critical for achieving sustainable reductions in the burden of NCDs across the region.

## Data Availability

The original contributions presented in the study are included in the article/supplementary material, further inquiries can be directed to the corresponding author.
